# Systematic review of Goal Attainment Scaling as an outcome measure in drug trials

**DOI:** 10.1186/1745-6215-16-S1-P14

**Published:** 2015-05-29

**Authors:** Charlotte M W Gaasterland, Marijke C Jansen-van der Weide, Johanna H van der Lee

**Affiliations:** 1 Pediatric Clinical Research Office Woman-Child Center, Academic Medical Center, University of Amsterdam, Meibergdreef 9, 1105 AZ Amsterdam, The Netherlands

## Introduction

Goal Attainment Scaling (GAS)[1] is a technique aimed to measure change induced by treatment. GAS enables patients to set goals and to determine the relative success in achieving these goals on a 5-point scale that is precisely defined beforehand. Since the goals are individually determined, goals may differ in actual content. Its individual and patient oriented approach is one of the appealing aspects of GAS when used as an outcome measure for trials, particularly in orphan diseases. GAS may be more responsive than standardised questionnaires, making it useful in smaller and more heterogeneous samples. In this systematic review, we aim to investigate whether the measurement properties of GAS have been evaluated in drug trials.

## Methods

We have conducted a sensitive search in Medline, PsycINFO and Embase. Included are papers that either describe a study in which a drug intervention is tested using GAS as an outcome measure, or in which the measurement characteristics of GAS are evaluated, in terms of validity, reliability, responsiveness, and/or feasibility. Selection, data extraction and critical appraisal is performed by 2 independent reviewers.

## Results

The search yielded a total of 3271 abstracts after removal of duplicates. Of those abstracts, 296 were assessed for eligibility. The final results of this review will become available in 2015.

## Conclusion

GAS may be a useful personalised approach to outcome measurement in rare conditions. We expect that further validation of the method will be needed.
